# The Effect of Intra-Arterial Angiotensin II on the Hepatic Tumor to Non-Tumor Blood Flow Ratio for Radioembolization: A Systematic Review

**DOI:** 10.1371/journal.pone.0086394

**Published:** 2014-01-17

**Authors:** Andor F. van den Hoven, Maarten L. J. Smits, Charlotte E. N. M. Rosenbaum, Helena M. Verkooijen, Maurice A. A. J. van den Bosch, Marnix G. E. H. Lam

**Affiliations:** Department of Radiology and Nuclear Medicine, University Medical Center Utrecht, Utrecht, The Netherlands; Osaka University Graduate School of Medicine, Japan

## Abstract

**Purpose:**

Treatment efficacy of intra-arterial radioembolization for liver tumors depends on the selective targeting of tumorous tissue. Recent investigations have demonstrated that tumors may receive inadequate doses of radioactivity after radioembolization, due to unfavorable tumor to non-tumor (T/N) uptake ratios of radioactive microspheres. Hepatic arterial infusion of the vasoconstrictor angiotensin II (AT-II) is reported to increase the T/N blood flow ratio. The purpose of this systematic review was to provide a comprehensive overview of the effect of hepatic arterial AT-II on T/N blood flow ratio in patients with hepatic malignancies, and determine its clinical value for radioembolization.

**Methods:**

This review was conducted in accordance with the Preferred Reporting Items for Systematic Reviews and Meta-Analyses guidelines. A structured search was performed in the PubMed, EMBASE and Cochrane databases. Only studies that presented data on T/N ratios before and after infusion of AT-II into the hepatic artery, in human patients with hepatic malignancies, were selected. Median T/N ratios before, during and after AT-II infusion, and the median T/N ratio improvement factor were extracted from the selected articles. All data on systemic blood pressure measurements and clinical symptoms were also extracted.

**Results:**

The search identified 524 titles of which 5 studies, including a total of 71 patients were considered relevant. Median T/N ratios before infusion of AT-II ranged from 0.4 to 3.4. All studies observed a substantial improvement of the T/N ratio after AT-II infusion, with median improvement factors ranging from 1.8 to 3.1. A transitory increase of systemic blood pressure was observed during AT-II infusion.

**Conclusions:**

Infusion of AT-II into the hepatic artery leads to an increase of the tumor to non-tumor blood flow ratio, as measured by T/N uptake ratios. Clinical trials are warranted to assess safety aspects, optimal administration strategy and impact on treatment efficacy during radioembolization.

## Introduction

Intra-arterial radioembolization (RE) with yttrium-90 (^90^Y-) microspheres has proven to be an effective treatment option for patients with unresectable primary or metastatic liver tumors, refractory to chemotherapy.[1], [2], [3], [4], [5] During this minimally-invasive therapy, a percutaneous approach is used to place a catheter in the hepatic artery and administer high-energy β-radiation emitting ^90^Y-microspheres that will irradiate liver tumors from within. This therapy thrives on the fact that liver tumors are primarily vascularized by the hepatic artery, while normal liver parenchyma receives the majority of its blood supply from the portal vein.[6] In theory, this should translate into a higher concentration of microspheres within the tumorous liver tissue than in healthy liver tissue, i.e. a favorable (>1) tumor to non-tumor (T/N) uptake ratio of radioactive microspheres.

Single-photon emission tomography - computed tomography (SPECT-CT) imaging, routinely performed after the arterial administration of ^99m^Tc-labelled macroalbumin aggregates (^99m^Tc-MAA) to estimate the fraction of liver-to-lung shunt and exclude potential extrahepatic shunting prior to RE, has been used to predict individual T/N microsphere uptake ratios based on the intrahepatic distribution of ^99m^Tc-MAA. Several studies reported that favorable T/N uptake ratios of ^99m^Tc- MAA are associated with improved post-treatment tumor response[7], [8], [9], whereas other studies contradict these findings and emphasize that assuming an equal intrahepatic distribution of ^99m^Tc-MAA and ^90^Y-microspheres may not be justified.[10], [11], [12], [13] Still, a consistent finding of these studies is a strong interindividual heterogeneity in T/N uptake ratio’s, with a reported range of 0.6–25.9.[7], [8], [9], [11], [12], [14], [15], [16], [17] Furthermore, a recent study assessing the radioactive microsphere biodistribution on post-treatment quantitative imaging following radioembolization, demonstrated that the T/N uptake ratio per tumor may also show marked variability within the same liver. Up to 60% of the patients had at least one tumor that received a concentration of radioactivity equal to or lower than the normal liver tissue (T/N ≤1). Thus, some tumors receive below therapeutic doses of radioactivity, whereas healthy liver tissue may receive toxic doses of radioactivity, resulting in suboptimal treatment[18]. The heterogeneity in T/N uptake ratios is a result of various factors including differences in tumor angiogenesis, microsphere characteristics and flow-bound distribution physics [9].

A common feature among liver tumors is that the vasculature of liver tumors is immature, lacking normal neurovascular innervation and a well-developed smooth muscle coating [19], [20], [21]. This allows for manipulation of the fraction of blood flowing to tumorous liver tissue (T/N blood flow ratio) by infusion of a vasoconstrictive agent. As opposed to the vasculature of the healthy liver tissue, tumor vessels will not respond to infusion of vasoconstrictive drugs, leading to an increase in preferential blood flow to the tumorous liver tissue. Various vasoconstrictors, including epinephrine, noradrenaline and angiotensin-II have shown the potential to increase the hepatic T/N blood flow ratio, in both preclinical and clinical studies [19], [22], [23], [24], [25]. Angiotensin II (AT-II) is an octapeptide hormone that acts as a vasoactive agonist and induces arterial vasoconstriction[26], with the most pronounced effect in the splanchnic vasculature.[24] Therefore, hepatic arterial AT-II infusion during RE seems a promising method to increase the T/N uptake ratio of radioactive microspheres.[27].

Previously, various clinical studies have reported the routine use of AT-II infusion during ^90^Y-RE [2], [4], [12], [28], [29], [30]. However, the efficacy of using AT-II for this purpose is not clear. Therefore, we performed a systematic review of the available evidence on the effect of intra-arterial AT-II infusion on the hepatic arterial T/N blood flow ratio in human patients with hepatic malignancies, weighing potential benefits for RE against expected side-effects.

## Methods

**Figure 1:**
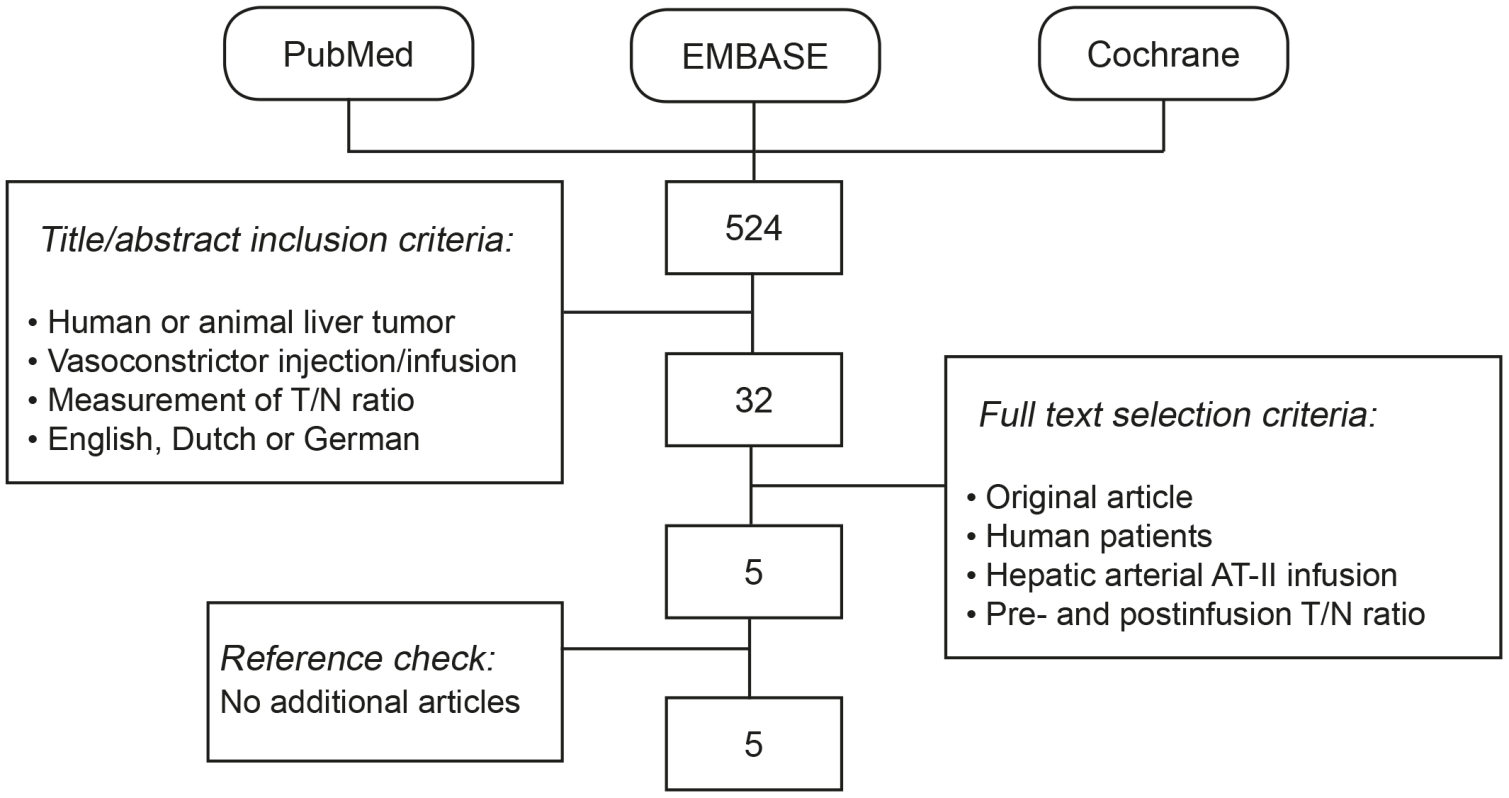
Flowchart illustrating the article selection process.

This systematic review was conducted according to the guidelines of the Preferred Reporting Items for Systematic Reviews and Meta-Analyses (PRISMA) Statement [31]. A structured search was performed in PubMed, EMBASE and Cochrane databases on February 28^th^ 2013. Various synonyms for vasoconstrictors were combined with synonyms for blood flow and liver (Table 1). The search results were screened for potentially relevant articles on title/abstract and full-text using predefined inclusion and selection criteria. To improve comparability between different studies, only articles that presented data on T/N ratios before and after infusion of AT-II into the hepatic artery in human patients with liver malignancies, were included. Cross check of reference lists from identified articles was performed to identify additional articles (Fig. 1).

**Table 1:** Search Syntax.

(The search was performed on 28-02-2013)
**PubMed** (442 results)
*(angiotensin OR noradrenaline OR vasopressin OR vasoconstrictor) AND (“blood-flow” OR “blood flow”) AND (liver OR hepatic)*
Limit: title/abstract
**EMBASE** (500 results)
*(angiotensin:ab,ti OR noradrenaline:ab,ti OR vasopressin:ab,ti OR vasoconstrictor:ab,ti) AND (‘blood-flow’:ab,ti OR ‘blood flow’:ab,ti) AND (liver:ab,ti OR hepatic:ab,ti)*
**Cochrane** (35 results)
*(angiotensin OR noradrenaline OR vasopressin OR vasoconstrictor) AND (“blood-flow” OR “blood flow”) AND (liver OR hepatic)*
In Cochrane Central Register of Controlled Trials (Clinical Trials)

In this table, the search syntax used in PubMed, EMBASE and Cochrane databases is displayed. The number of results include double titles. In total, 524 original titles were identified.

Subsequently, a quality assessment of the selected articles was performed with the use of the Strengthening the Reporting of Observational Studies in Epidemiology (STROBE) statement [32]. All articles were assessed according to the following criteria: clearly described inclusion criteria, presence of a control group, standardization of the AT-II administration, standardization of the T/N ratio measurement and blinding for the outcome measurement.

Two reviewers (AH, CR) independently extracted the following data from included studies: the size of study population, tumor type, AT-II dose, method for estimating the T/N blood flow ratio, median T/N ratios before and after administration of AT-II, and median improvement factor. Reported mean T/N ratios were converted to median values to account for non-normality and improve the comparability of the results. Reported median improvement factors indicated the median factor of improvement in T/N ratio induced by the infusion of AT-II, as calculated according to the following formula:
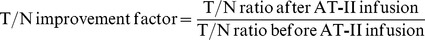



Hypertension is a well-known side effect of intra-arterial AT-II infusion. Therefore, we also extracted all data on systemic blood pressure measurements and clinical symptoms due to AT-II infusion. Discrepancies in extracted data were resolved by consensus, after revision of the reported data.

## Results

### Search Results

The search identified 524 original titles (Fig. 1). After screening the articles on title/abstract, and subsequently reading 32 articles in full-text, five relevant articles were included. Two selected articles were published by Goldberg *et al*. in the same year and journal, and are referred to as Goldberg *et al.* 1 and Goldberg *et al.* 2 to avoid confusion.

### Quality Assessment and Study Characteristics

The quality assessment scores of the selected studies are listed in Table 2. All selected studies were before-and-after studies. The inclusion criteria were not clearly described in 4/5 (80%) studies. None of the studies used a control group, and the measurement of the outcome was not performed in a blinded fashion. All studies standardized the AT-II administration and T/N ratio measurement.

**Table 2:** Quality assessment of selected studies.

Author (year)	Inclusion criteria clearly described	Controlgroup	Standardization of AT-II administration	Standardization of T/N ratio measurement	Blinding for outcome measurement
**Sasaki ** ***et al.*** [**25**]	N	N	Y	Y	N
(1985)					
**Goldberg ** ***et al.*** ** 1** [**33**]	N	N	Y	Y	N
(1991)					
**Goldberg ** ***et al.*** ** 2** [**34**]	N	N	Y	Y	N
(1991)					
**Burke ** ***et al.*** [**19**]	Y	N	Y	Y	N
(2001)					
**Ishikawa ** ***et al.*** [**36**]	N	N	Y	Y	N
(2007)					

Abbreviations: Y = Yes, N = No.

### Study Methods and Patients

The characteristics of the selected studies are summarized in Table 3.

**Table 3:** Characteristics of studies on the effect of AT-II on the T/N ratio in patients with hepatic malignancies.

Author (year)	Study size*N patients*(tumors)	Tumor type(N patients)	AT-IIdose	Radioactivetracer	T/Nmeasurement
**Sasaki ** ***et al.*** [**25**]	9 (14)	HCC (7)	10 μg/min for 3–4 min	^81m^Kr solution	RA measurement with scintillation camera during infusion
(1985)		CRLM (2)			
**Goldberg ** ***et al.*** ** 1** [**33**]	9	CRLM (8)	10 μg/min for 100 sec	^131^I before and	Biopsies of tumor and non-tumor. RA measured with
(1991)		UP (1)		^99m^Tc after AT-II	scintillation camera
**Goldberg ** ***et al.*** ** 2** [**34**]	9 (48)	CRLM (9)	10 μg/min for 100 sec	^99m^Tc MSA	SPECT after albumin colloid injection and HAPS
(1991)					
**Burke ** ***et al.*** [**19**]	9 (11)	CRLM (9)	5 μg/min for 45 min	^62^Cu-PTSM	PET
(2001)					
**Ishikawa ** ***et al.*** [**36**]	35	PCLM (35)	5 μg/min for 10 sec	None	CT contrast enhancement measurement
(2007)					

Abbreviations: HCC = Hepatocellular Carcinoma. CRLM = Colorectal Liver Metastases. UP = Metastases of an Unknown Primary. PCLM = Pancreatic Cancer Liver Metastases. NA = Not Available. RA = radioactivity. SPECT = Single-Photon Emission Computed Tomography. HAPS = Hepatic Arterial Perfusion Scintigraphy. PET = Positron Emission Tomography. ^81m^Kr = Krypton-81m. ^131^I = Iodine-131. ^99m^Tc MSA = Radiolabeled albumin microspheres. ^62^Cu-PTSM = copper-pyruvaldehyde-bis (N4-methylthiosemicarbazone).

Sasaki *et al.* [25] investigated AT-II induced changes in the distribution of the hepatic blood flow in a total of nine patients (14 tumors), including seven patients with hepatocellular carcinoma (HCC, nine tumors) and two patients with colorectal liver metastases (CRLM, five tumors). Radioactivity was measured with a scintillation camera placed over the liver during a continuous infusion of a short-lived ^81m^Kr-solution (half-life 13 seconds). The planar images of the liver were then played back on a color display according to the intensity of radioactivity. ^81m^Kr-images and images of a colloid hepatic scintigram were superimposed on the color display. Subsequently, regions of interest (ROIs) were drawn both on tumor region, indicated by defects in a baseline ^99m^Tc-stannum colloid hepatic scintigram, and non-tumor region. The radioactivity per frame was calculated for the ROI sets, and the effect of AT-II on the T/N ratio was evaluated by comparing the radioactivity in the tumor and non-tumor region before, and after a hepatic arterial infusion of 10 μg/min AT-II for 3–4 minutes. In one patient, different AT-II administrations were compared to determine the optimum administration strategy: hepatic arterial infusion of 5 μg/min, hepatic arterial infusion of 10 μg/min and intravenous infusion of 5 μg/min. It was found that hepatic arterial infusion at a rate of 10 μg/min resulted in higher T/N ratios when compared to using a rate of 5 μg/min, and did not increase systemic blood pressure as much as an intravenous infusion.

Goldberg *et al.* 1[33] used a double isotope method to assess the effect of AT-II on the distribution of radiolabeled microspheres in nine patients with liver metastases, including eight patients with CRLM and one patient with an unknown primary. The patients underwent a laparotomy under general anesthesia for the placement of a hepatic arterial catheter for regional chemotherapy. First, a tracer dose of ^131^I- microspheres was injected. Subsequently, AT-II was infused into the catheter at a rate of 10 μg/min for 100 seconds. Immediately after infusion of AT-II, a tracer dose of ^99m^Tc-microspheres was given. Biopsies of tumor and adjacent normal liver tissue were obtained and weighed, and the uptake of ^131^I- and ^99m^Tc-microspheres in tumor and non-tumor tissue was determined by measuring radioactivity with a gamma scintillation counter. T/N ratios were calculated by comparing the relative microsphere content of the tissue samples.

In another study, Goldberg *et al.* 2[34] used scintigraphic planar and tomographic imaging to determine the effect of AT-II on the distribution of radiolabeled albumin microspheres in nine patients with CRLM (48 tumors). Patients received a SPECT scan at three different moments: 1) after intravenous injection of albumin colloid (^99m^Tc-albumin colloid) to localize the metastatic tumors, 2) after injection of radiolabeled albumin microspheres (^99m^Tc-MSA) prior to the infusion of AT-II, and 3) after injection of ^99m^Tc-MSA, immediately after the hepatic arterial infusion of AT-II at a rate of 10 μg/min for 100 seconds. All studies were performed in random order, but at least three days apart and were completed within two weeks. A tomographic gamma camera was used, and the planar anterior tomographic acquisitions were reconstructed to transverse slices. After superimposing the albumin colloid study and both microsphere studies, ROIs were drawn to define tumor (regions of low uptake on the albumin colloid scan) and normal liver areas (a sampled area of uniform high uptake on the albumin colloid scan). T/N ratios were calculated before and after the infusion of AT-II, by comparing the activity within both areas. Two patients with markedly hypervascular lesions (T/N ratios >30 before AT-II administration) were non-assessable, because it was not possible to obtain sufficient counts in the normal liver, irrespective of the AT-II administration.

Burke *et al.* [19] used Positron Emission Tomography (PET) after administration of the short-half-life positron emitter copper-pyruvaldehyde-bis (N^4^-methylthiosemicarbazone) (^62^Cu-PTSM) to study the effect of AT-II on the T/N ratio in nine patients with CRLM (11 tumors). A baseline PET scan with ^62^Cu-PTSM was compared to two additional PET scans with ^62^Cu-PTSM: one during (10 minutes after the start of AT-II infusion) and one immediately after completion of a continuous hepatic arterial infusion of AT-II at a rate of 5 μg/min for 45 minutes. In one patient with CRLM, AT-II had been substituted for physiological saline to confirm the reproducibility of hepatic arterial blood flow distribution measurements with ^62^Cu-PTSM PET [35].

Ishikawa *et al* [36] used CT angiography to assess the effect of AT-II on the T/N blood flow ratio in 35 patients with pancreatic cancer liver metastases (PCLM). Median attenuation values of ROIs in tumor and normal liver parenchyma were measured on CT angiography after hepatic arterial infusion of AT-II at a rate of 5 μg/min for 10 seconds, and compared to values on a baseline scan.

### Tumor to Non-tumor Blood Flow Ratios

Median T/N ratios before infusion of AT-II ranged from 0.4 to 3.4. Median T/N ratios after administration of AT-II ranged from 0.8 to 7.3. All studies observed an improvement of the T/N ratio after AT-II infusion with median improvement factors ranging from 1.8 to 3.1. The T/N ratio before and after infusion of AT-II, and the T/N improvement factors observed in each study are summarized in Table 4.

**Table 4:** T/N ratios before, during and after AT-II infusion, and T/N ratio improvement factors in patients with hepatic malignancies.

Author (year)	T/N ratiopre-infusion	T/N ratio during infusion	T/N ratio post-infusion	T/N improvement factor
**Sasaki ** ***et al.*** [**25**]	median 1.36	-	median 4.05	median 3.07 (1.46–5.33)
(1985)	(0.38–3.36)		(1.56–9.33)	*p*<0.001
**Goldberg ** ***et al.*** ** 1** [**33**]	median 0.36	-	median 0.75	median 2.80 (0.80–11.70)
(1991)	(0.11–0.90)		(0.10–8.11)	*p*<0.05
**Goldberg ** ***et al.*** ** 2** [**34**]	median 3.4	-	median 7.3	median 1.8 (0.5–3.4)
(1991)	(1.3–6.0)		(1.5–8.8)	*p*<0.05
**Burke ** ***et al.*** [**19**]	median 1.3	median 2.1	median 1.85	NR
(2001)	(0.93–6.21)	(0.63–22.86)	(0.70–18.34)	*p* = 0.008 and p = 0.03*
**Ishikawa ** ***et al.*** [**36**]	median 1.03	-	median 1.34	NR
(2007)	(0.41–1.72)		(0.84–2.09)	*p* = 0.01

T/N ratios are described as median (range). P-values correspond to reported significance levels of Wilcoxon test for paired data. *The first p-value refers to the improvement of T/N ratio during infusion and the last to post-infusion. Abbreviations: NR = Not Reported.

Sasaki *et al* [25] observed a substantial (>1.5-fold) increase of the T/N ratio after AT-II, in all nine patients and all fourteen tumors. The T/N ratio increased from a median value of 1.36 (range 0.38–3.36) before AT-II to 4.05 (1.56–9.33) after the administration of AT-II. Furthermore, the continuous radioactivity measurement in this study revealed that the T/N ratio reached a peak at approximately 100 seconds after the infusion of AT-II, and decreased gradually afterwards.

Goldberg *et al.* 1[33] found that the uptake of microspheres increased in seven patients (78%), and slightly decreased in two (22%) patients. The median T/N ratio before the administration of AT-II increased from 0.36 (0.11–0.90) to 0.75 (0.10–8.11) afterwards.

Goldberg *et al.* 2[34] also found that the uptake of radiolabeled albumin microspheres increased in seven patients (78%), and slightly decreased in two (22%) patients. In this study, the T/N ratio increased from a median value of 3.4 (1.3–6.0) before AT-II to 7.3 (1.5–8.8) after the administration of AT-II.

Burke *et al.* [19] determined the T/N ratio during (10 minutes after the start) and after completion of a prolonged AT-II infusion of 45 minutes. During the infusion, the T/N ratio was increased in 7/9 (78%) patients and 7/11 (63%) lesions. At the end of the AT-II infusion, the T/N ratio was still increased in 6/9 (67%) patients and 6/11 (55%) lesions. The median T/N ratio increased from a baseline value of 1.3 (0.93–6.21) to 2.1 (0.63–22.86) during and to 1.85 (0.70–18.34) after the prolonged AT-II infusion.

Ishikawa *et al.* [36] observed an AT-II induced increase in median T/N ratio from 1.03 (0.41–1.72) before the administration of AT-II to 1.34 (0.84–2.09) afterwards.

### Adverse Effects

All studies observed a transitory AT-II induced increase in systemic blood pressure (BP). After the infusion, blood pressure gradually decreased towards baseline levels.

Sasaki *et al.* [25] observed an 1.5-fold increase from 124 mm Hg (± standard deviation 31) at baseline to 186 mm Hg (±40). Within three minutes after ceasing the AT-II infusion, BP had returned to baseline in all patients. In one patient, different administration strategies were employed. In this patient, hepatic arterial infusion of AT-II did not increase systemic blood pressure as much as intravenous infusion.

Goldberg *et al.* 1[33] found a maximal increase of 40 mm Hg in systolic BP at the end of the 100 s AT-II infusion with a gradual decrease afterwards.

Burke *et al.* [19] observed an increase in median arterial blood pressure from 98 mm Hg (interquartile range 97–108) at baseline to 114 mm Hg (interquartile range 109–115) after 10 min of AT-II infusion. A substantial decrease towards baseline levels occurred in all cases within a few minutes after cessation of the infusion. They did not observe changes in pulse rate.

Ishikawa *et al.* [36] found an increase in systemic BP of up to 40 mm Hg in systolic blood BP. When blood pressure was elevated, patients complained of a sense of chest oppression and headache, but these symptoms were not severe enough to hamper the administration of AT-II. No other adverse effects were reported.

## Discussion

We systematically reviewed the available evidence on the effect of intra-arterial AT-II infusion on the hepatic T/N blood flow ratio in patients with hepatic malignancies to determine its clinical value for RE. All studies showed a substantial increase of the T/N ratios after infusion of AT-II into the hepatic artery. Median improvement factors of the T/N ratio ranged from 1.8 to 3.1.

Whole liver external beam irradiation, instead of RE, is of limited value in the treatment of liver tumors, due to the healthy liver tissue’s low tolerance for ionizing radiation.[37] This lead to the development of selective internal radiation therapy. During RE, the predominant hepatic arterial vascularization of liver tumors enables the preferential delivery of radioactive microspheres to tumorous tissue, resulting in very high radiation doses in the tumors while the healthy liver tissue radiation exposure remains within tolerable limits.[7], [11], [38] Pathological examinations of livers treated with RE have revealed that, following infusion, ^90^Y-microspheres are preferentially deposited in clusters within the tumor periphery, with 200-times greater microsphere concentrations than in the tumor center and the healthy liver tissue.[14], [38] Perfusion scintigraphy has been used to quantify this tumor selectivity, by calculating the T/N uptake ratio of ^99m^Tc-MAA. These studies have revealed a strong heterogeneity in the T/N ratio, with reported values ranging from 0.6–25.9.[7], [8], [9], [11], [12], [14], [15], [16], [17] The fact that the hepatic arterial T/N blood flow ratio is highly variable between patients and between different tumors within the same patient, poses a clinical problem. Kao *et al.* noted that in their study in patients with inoperable HCC, the SPECT/CT based median intrapatient difference of the ^99m^Tc-MAA T/N uptake ratio was 1.9 (95% confidence interval 1.1–2.5).[17] Furthermore, Flamen *et al.* reported that up to 38% of the metastatic liver lesions in their study had an unfavorable ^99m^Tc-MAA T/N uptake ratio (<1). The authors advocated the use of a ^99m^Tc-MAA T/N uptake ratio >1 as a patient selection criterion, because it was demonstrated that this was strongly predictive of metabolic post-treatment response on PET/CT. In patients with a T/N uptake ratio <1, tumors may receive inadequate doses of radioactivity, while the radioactive burden on the healthy liver tissue may exceed toxic levels. Using an intra-arterial AT-II infusion during RE may prove a way to overcome the problem of unfavorable T/N ratios, and optimize treatment-efficacy. Various studies have found an association between tumor absorbed radiation doses, tumor response and overall survival.[39], [40], [41], [42] It can, therefore, be expected that improved tumor selectivity of radioactive microsphere distribution will positively affect patient outcome after RE.

The selected studies showed consistent and promising effects of AT-II on the hepatic arterial T/N blood flow ratio. There are, however, some limitations to these studies. First, all selected studies were small non-controlled studies, prone to selection and observer bias. Second, patients with different tumor types were included in the selected studies. The majority of the studies included patients with colorectal liver metastases. However, Ishikawa *et al.* [36] only included patients with liver metastases from pancreatic cancer, and the majority of patients in Sasaki’s study had HCC.[25] Angiogenesis of liver tumors is a complex process, and the degree of arterial vascularization is dependent on multiple factors, including the stage of development, grade of malignancy, tumor size and origin of the primary tumor. Early stage well-differentiated hepatocellular carcinoma (HCC) for example, receives both arterial and portal blood supply through small unpaired arteries and portal tracts within the tumor. However, during growth of the tumor, portal tracts will be deformed and decrease in number, and arterial tumor vessels enter the tumor. Once the tumor is encapsulated, the advanced HCC is exclusively vascularized by arterial tumor vessels.[43] The vascularity of liver tumors is characterized by their contrast enhancement in the arterial phase on computed tomography (CT) or magnetic resonance imaging (MRI).[44] HCC is in general hypervascular, but metastatic liver tumors can be either hypovascular, hypervascular or display a more complex pattern of enhancement. Therefore, one could argue that hypervascular tumors like HCC have different hemodynamic characteristics than hypovascular liver metastases and may respond differently to the infusion of AT-II. Hence, it is interesting to note that the study of Sasaki *et al*.[25] (HCC) showed the greatest improvement in T/N ratio after AT-II infusion.

Third, it is currently very challenging to perform direct and accurate measurements of the differential arterial blood flow to liver tumors and normal liver parenchyma. Therefore, AT-II induced changes in the T/N blood flow ratio need to be estimated by using other methods such as measurements of radioactive tracer/microsphere uptake or contrast-enhancement on CT-imaging. Several challenges need to be overcome in order to avoid biased or inaccurate estimations. T/N ratios should be determined as the total amount of a certain agent (i.e. radioactive tracer, contrast) in the total volume of all tumors (tumorous tissue volume) divided by the amount of that agent in the total liver volume minus the tumor volume (i.e. healthy liver volume). Selecting an area of tumorous and non-tumorous tissue by drawing ROIs on planar scintigraphic imaging, or taking a biopsy of both areas may be highly subjective and introduce bias. In addition, distinguishing healthy liver from viable tumorous tissue and registering that spatial information with activity distribution is a challenging task. The methods in the selected studies are somewhat outdated. The two studies that used colloid hepatic scintigraphy to identify tumorous liver tissue[25], [34], superimposed planar images of the colloid scans with those acquired after the administration of radioactive tracers. This may result in malregistration of images. Burke *et al.* [19] on the other hand, did not fuse PET-images with the anatomical CT-images, which in turn may result in incorrect localization of tumors.[45] These problems may be overcome by using hybrid imaging that combines functional and anatomical information, such as ^18F^FDG-PET-CT, and allows for the definition of three-dimensional volumes of interest (VOIs). Furthermore, dual tracer imaging with ^99m^Tc-MAA-^99m^Tc-sulfur colloid SPECT [46] and dynamic magnetic resonance imaging with the hepatobiliary specific contrast agent gadoxetic acid (Primovist, Bayer-Schering, Berlin, Germany)[47] are two relatively new functional imaging strategies that may prove to be particularly useful to distinguish tumorous from healthy liver tissue.

To determine the potential of using AT-II for the purpose of RE, the effect on the T/N uptake ratio of radioactive microspheres should be assessed. The technological developments of the last decades enable us to perform quantitative studies on the biodistribution of microspheres. Dosimetry post-^90^Y-RE, for example, used to be solely based on SPECT of indirectly generated gamma-photons (Bremsstrahlung). Post-treatment imaging with PET, based on the internal pair production of ^90^Y once every 32 million decays, has substantially improved the post-treatment quantification of ^90^Y-microspheres.[48], [49], [50] Furthermore, our institute has developed holmium-166 (^166^Ho) poly (L-lactic acid) microspheres to allow for quantitative imaging of microsphere distribution.[51], [52] In addition to high-energy beta radiation, ^166^Ho also emits gamma-radiation and thereby facilitates imaging by gamma scintigraphy and SPECT.[53], [54] Moreover, the distribution of ^166^Ho-microspheres can be visualized by MRI due to its highly paramagnetic properties.[54], [55], [56] The overall evidence for the use of AT-II to improve T/N microsphere uptake ratios would benefit from additional studies trying to confirm these findings, using the above-mentioned imaging techniques for dosimetry.

When AT-II is used in clinical practice, it may lead to a sudden rise in systemic blood pressure. In theory, this could be accompanied by serious side effects. We therefore screened the selected articles on any side effects of AT-II infusion. All studies that monitored systemic blood pressure observed a transitory increase in systolic blood pressure due to the AT-II infusion. Blood pressure returned to baseline levels within a few minutes after the infusion was stopped, and no relevant increase of heart rate was observed. Remarkably, only one study[36] reported on symptoms experienced by patients due to the AT-II infusion. In this study, symptoms were not severe and all transient.

Infusion of AT-II during administration of microspheres may also influence the total amount of microspheres that can be delivered before stasis occurs. This can be particularly relevant for treatment with resin-based ^90^Y-microspheres, since early arterial stasis and subsequent inability to deliver the whole prescribed dose has been reported in up to 20% of cases.[57] Although no early stasis was reported in the reviewed studies, this matter deserves further study using ^90^Y-microspheres.

AT-II infusion duration and doses differed between studies and the optimal infusion strategy of AT-II remains uncertain. An infusion time of 100 seconds seems justified, since in one study[25] T/N ratios were continuously measured during a 3–4 infusion period of AT-II and T/N ratios were highest after 100 seconds of infusion. Furthermore, the two studies[33], [34] that used a 100 seconds infusion time had better T/N ratio improvement factors than the 10 seconds[36] and 45 minutes studies[19]. One study[25] compared different infusion strategies in one patient and concluded that the improvement in T/N ratio was greater with a hepatic arterial infusion of 10 μg AT-II per minute, compared to 5 μg/min. AT-II has already been used routinely as part of the treatment protocol of randomized controlled trials and non-randomized studies investigating the effect of intra-arterial RE with ^90^Y-microspheres.[2], [4], [12], [28], [29], [30] These studies used a hepatic arterial bolus injection of 25 or 50 μg AT-II shortly (approximately 30 seconds) before the administration of ^90^Y-microspheres. Unfortunately, none of these studies reflected on the toxicity or efficacy of the intra-arterial AT-II infusion. A comparative study (ideally a placebo-controlled randomized trial) should determine the impact of AT-II administration on patient outcome after RE.

## Conclusion

Infusion of AT-II into the hepatic artery leads to an increase of the tumor to non-tumor blood flow ratio, as measured by T/N uptake ratios. Clinical trials are warranted to assess safety aspects, optimal administration strategy and impact on treatment efficacy during radioembolization.

## Supporting Information

Checklist S1 (DOC)
